# Effects of probiotics on blood lipids, glucose and pressure in patients with coronary heart disease: a systematic review and meta-analysis

**DOI:** 10.3389/fcvm.2026.1707408

**Published:** 2026-02-06

**Authors:** Yan Zhong, Xinyu Yang, Weiming Kong, Yaru Shi, Weixiong Jian

**Affiliations:** 1 School of Traditional Chinese Medicine, Hunan University of Chinese Medicine, Changsha, Hunan, China; 2 The First Clinical College of Chinese Medicine, Hunan University of Chinese Medicine, Changsha, Hunan, China; 3 Provincial Key Laboratory of TCM Diagnostics, Hunan University of Chinese Medicine, Changsha, Hunan, China

**Keywords:** coronary heart disease, meta-analysis, probiotics, randomized controlled trials, trial sequential analysis

## Abstract

**Objective:**

This study aims to investigate the effects of probiotic supplementation on blood glucose, lipids and pressure in patients with coronary heart disease (CHD) through systematic review and meta-analysis, combined with sequential trial analysis, and to assess its safety.

**Methods:**

A systematic search was conducted across five English-language databases: PubMed, Embase, Cochrane Library, Web of Science, and MEDLINE. The search period spanned from the inception of each database to May 31, 2,025. The baseline characteristics and data from the included studies were extracted and analyzed. Meta-analysis and trial sequential analysis (TSA) were performed using RevMan 5.3 and TSA 0.9.5.10 beta, respectively.

**Results:**

A total of nine studies involving 478 patients were included in this meta-analysis. The pooled results demonstrated that, compared with the placebo group, probiotic supplementation significantly reduced levels of low-density lipoprotein cholesterol (LDL-C) [mean difference [MD] −11.16, 95% confidence interval [CI] −18.82 to −3.50, *P* = 0.004], total cholesterol (TC) (MD −9.32, 95% CI −18.01 to −0.63, *P* = 0.04), fasting blood glucose (FBG) (MD −7.82, 95% CI −15.60 to −0.04, *P* = 0.05), and insulin (MD −2.47, 95% CI −4.16 to −0.78, *P* = 0.004), and increased high-density lipoprotein cholesterol (HDL-C) levels (MD 2.24, 95% CI 0.61 to 3.87, *P* = 0.007). No significant effects were observed on very-low-density lipoprotein cholesterol (VLDL-C) (MD −2.89, 95% CI −6.83 to 1.05, *P* = 0.15), triglyceride (TG) (MD −13.45, 95% CI −28.60 to 1.70, *P* = 0.08), homeostasis model assessment of insulin resistance (HOMA-IR) (MD −0.43, 95% CI −1.13 to 0.28, *P* = 0.23), QUICK (MD 0.00, 95% CI −0.00 to 0.01, *P* = 0.25), systolic blood pressure (SBP) (MD −1.99, 95% CI −4.97 to 1.00, *P* = 0.19), diastolic blood pressure (DBP) (MD −1.23, 95% CI −3.32 to 0.86, *P* = 0.25), or the incidence of adverse events (MD 2.00, 95% CI 0.20 to 20.49, *P* = 0.56). Trial sequential analysis confirmed that the evidence for LDL-C and insulin was sufficient to reach firm conclusions.

**Conclusion:**

Probiotics have been shown to significantly reduce LDL-C and insulin levels in patients with CHD without increasing the risk of adverse events. However, the impact of probiotics on other metabolic parameters such as TC, FBG, and HDL-C remains inconclusive and requires further investigation through well-designed studies.

**Systematic Review Registration:**

https://www.crd.york.ac.uk/PROSPERO/ PROSPERO CRD420251272111.

## 1 Introduction

Coronary heart disease (CHD), also referred to as coronary atherosclerotic heart disease, is characterized by chronic inflammation, lipid deposition, and the formation of atherosclerotic plaques as its underlying pathological mechanisms (1). The incidence of CHD rises with advancing age, with a notably increased risk observed in men aged 45 years or older and women aged 55 years or postmenopausal (2, 3). Hypertension, hyperlipidemia, and hyperglycemia are well-established as the three major modifiable risk factors for CHD (4). Studies have indicated that approximately 66.7% of deaths among patients with type 2 diabetes mellitus (T2DM) occur in those with concomitant CHD (5). Current standard pharmacological treatments for CHD include statins, beta-blockers, and angiotensin-converting enzyme inhibitors. Although these therapies have been shown to slow disease progression, their efficacy remains suboptimal, and concerns regarding long-term adverse effects persist (6–8). Therefore, there is a pressing need to identify and develop complementary therapeutic strategies to further enhance the clinical outcomes of CHD patients.

In recent years, the association between gut microbiota and CHD has emerged as a prominent area of research. Patients with CHD commonly exhibit gut microbial dysbiosis, characterized by reduced abundance of beneficial bacteria, overgrowth of pro-inflammatory species, and a marked decrease in microbial diversity (9). Among the metabolites produced by gut microbiota, trimethylamine N-oxide (TMAO), short-chain fatty acids (SCFAs), and lipopolysaccharide (LPS) have been most strongly implicated in the pathogenesis of CHD (10–12). Dysbiotic gut microbiota contributes to disease progression by promoting TMAO production, which activates inflammatory pathways and directly induces the transformation of macrophages into foam cells (13). Localized intestinal inflammation further compromises the integrity of the gut barrier, allowing LPS to enter systemic circulation and trigger the release of pro-inflammatory cytokines. These inflammatory mediators are subsequently transported via the bloodstream to the cardiovascular system, where they facilitate the development of atherosclerotic plaques (14). In contrast, SCFAs produced by probiotics can reinforce the intestinal barrier, thereby preventing the translocation of LPS into circulation (15), which in turn suppresses the progression of arteriosclerosis (AS) (16). Collectively, these findings highlight the close interplay between gut microbiota and CHD, suggesting that probiotics and their bioactive metabolites may hold therapeutic potential for improving CHD outcomes.

Probiotics are live microorganisms that confer health benefits to the host when administered in sufficient quantities (17). Although two previously published meta-analyses have preliminarily explored the effects of probiotics on glucose and lipid metabolism in patients with CHD (18, 19), both studies exhibit certain inherent limitations. For example, Dong et al. restricted their database search to PubMed, Embase, and Web of Science, and ultimately included only five eligible studies, which may have resulted in the omission of relevant literature (18). Lei et al. incorporated studies involving probiotics combined with trace elements, thereby introducing potential confounding variables, and did not assess the certainty of the evidence (19). In response to these limitations, this meta-analysis adopts a more comprehensive search strategy and more stringent exclusion criteria, with a specific focus on the impact of probiotic supplementation on blood glucose levels, lipid profiles, and blood pressure in CHD patients, aiming to strengthen the evidence base supporting the use of probiotics as an adjunctive therapy for CHD.

## 2 Method

This study was conducted in accordance with the Preferred Reporting Items for Systematic Reviews and Meta-Analyses (PRISMA) statement (20). Additionally, this systematic review was registered on the International Prospective Register of Systematic Reviews (PROSPERO), registration number [CRD420251272111].

### 2.1 Inclusion criteria

The inclusion criteria were formulated according to the PICOS framework: (i) participants (P): adult patients diagnosed with CHD; (ii) intervention (I): administration of standard CHD treatment combined with probiotic supplementation; (iii) control (C): administration of standard CHD treatment alone; (iv) outcomes (O): efficacy outcomes included low-density lipoprotein cholesterol (LDL-C), high-density lipoprotein cholesterol (HDL-C), very-low-density lipoprotein cholesterol (VLDL-C), total cholesterol (TC), triglyceride (TG), fasting blood glucose (FBG), Insulin, homeostasis model assessment of insulin resistance (HOMA-IR), and the quantitative insulin sensitivity check index (QUICKI), calculated as 1/[log(fasting insulin) + log(fasting glucose)], as well as systolic blood pressure (SBP), and diastolic blood pressure (DBP). LDL-C was designated as the primary efficacy outcome. Safety outcomes were assessed in terms of adverse event rates. (v) Study Type (S): randomized controlled trials (RCTs).

### 2.2 Exclusion criteria

(i) Duplicate publications or overlapping studies; (ii) Studies with incomplete or insufficient data; (iii) Studies lacking a placebo control group; (iv) Studies with evidence of selective outcome reporting.

### 2.3 Database search and search strategy

A systematic literature search was conducted independently by two reviewers across five English-language databases: PubMed, Embase, the Cochrane Library, Web of Science, and MEDLINE. The search covered the period from the inception of each database to May 31, 2025, without language restrictions. Searches were limited to the Title and Abstract fields, and the detailed search strategies are presented in Table 1. All records retrieved by the two reviewers were imported into EndNote ×9 software (Clarivate Analytics) for reference management. The numbers of records retrieved from each database were cross-checked by the two reviewers to ensure consistency and completeness of the search results.

**Table 1:** Search strategy.

Serial number	Subject terms	Search terms
#1	Probiotics	Probiotics OR Probiotic OR Bifidobacterium OR Bifidobacteria OR Bacillus bifida OR Yeast OR Saccharomyces cerevisiae OR Saccharomyces italicus OR Saccharomyces oviformis OR S cerevisiae OR S. cerevisiae OR Saccharomyces uvarum var melibiosus OR Candida robusta OR Saccharomyces capensis OR Lactobacillus acidophilus OR Lactobacillus amylovorus OR Lactobacill OR lactic acid bacteria OR Clostridium butyricum OR Bacillus OR Natto Bacteria OR Streptococcus thermophiles OR Enterococcus
#2	Coronary Heart Disease	Coronary Heart Diseases OR Coronary Disease OR Coronary Diseases OR Coronary Heart Disease OR Coronary Artery Disease OR Coronary Artery Diseases OR Myocardial Infarction OR Heart Attack OR Heart Attacks OR Myocardial Infarct OR Myocardial Infarcts
#3	–	#1 AND #2

### 2.4 Literature screening and data extraction

Literature screening and data extraction were conducted independently by two reviewers, with any disagreements resolved through discussion with a third independent reviewer (21). After duplicate removal using EndNote ×9 software (Clarivate Analytics), the remaining records were initially screened based on titles and abstracts to exclude obviously irrelevant studies. Full-text articles of potentially eligible studies were then retrieved and assessed for final inclusion according to the predefined inclusion and exclusion criteria based on the PICOS framework described in Section 2.1. The literature screening process did not impose a predefined start date; all studies published up to May 31, 2025, were considered for eligibility. Data extraction was conducted using a standardized data extraction form developed in Microsoft Excel 2010 (Microsoft Corporation) (22). The following information was extracted from each included study: author names, year of publication, country of origin, sample size, proportion of female participants, mean age, probiotic strain types, control interventions, intervention duration, and outcome measures.

### 2.5 Risk of bias assessment

The methodological quality of the included RCTs was independently assessed by two reviewers using the Cochrane Risk of Bias tool 1.0 (23). To ensure consistency, the results were cross-verified between the two reviewers. Any discrepancies were resolved through discussion with a third independent reviewer. Each study was evaluated across seven domains: random sequence generation, allocation concealment, blinding of participants and personnel, blinding of outcome assessment, incomplete outcome data, selective reporting, and other potential sources of bias. Each domain was categorized as having a low, high, or unclear risk of bias. This systematic approach ensured a rigorous and standardized evaluation of the methodological quality of the included studies.

### 2.6 Statistical analysis

Quantitative synthesis of outcome data was performed using RevMan 5.3 software. For dichotomous outcomes, the relative risk (RR) was employed as the effect measure, along with the calculation of the 95% confidence interval (CI). For continuous outcomes, the mean difference (MD) was selected as the effect measure, and the corresponding 95% CI was also computed. Heterogeneity across studies was assessed using the I² statistic. An I² value less than 50% was considered to indicate low heterogeneity, in which case a fixed-effect model was applied for pooling the effect sizes. Conversely, when I² exceeded 50%, suggesting substantial heterogeneity, a random-effects model was utilized (24). All hypothesis tests were two-tailed, and statistical significance was set at *P* < 0.05.

Funnel plots were employed to visually assess potential publication bias (25). In the absence of publication bias, the plot should exhibit a symmetrical inverted funnel shape. Asymmetry in the plot may indicate the presence of publication bias. It is important to note that when fewer than 10 studies are included, the statistical power of such tests may be limited, and the results should therefore be interpreted with caution.

For outcome indicators showing statistically significant results in the meta-analysis, trial sequential analysis (TSA) was further performed using TSA 0.9.5.10 beta software. The core procedures of TSA are as follows: (i) Based on a predefined type I error rate (*α* = 0.05), type II error rate (*β* = 0.20), and the pooled effect size from the included studies, the required information size (RIS) was calculated. RIS represents the minimum number of participants needed to reliably confirm or reject the observed intervention effect. (ii) A cumulative Z-curve was constructed, with the horizontal axis representing the cumulative sample size and the vertical axis representing the Z-statistic (26). The TSA monitoring boundary and RIS threshold were simultaneously marked on the plot. If the cumulative Z-curve crosses either the TSA boundary or the RIS threshold, sufficient evidence has been accumulated to support the reliability of the conclusion. Conversely, if the Z-curve does not cross any boundary and fails to reach the RIS threshold, additional studies are warranted to confirm the findings and prevent premature conclusions due to insufficient data.

### 2.7 Quality assessment of evidence

The quality of evidence for each outcome was assessed using the Grading of Recommendations Assessment, Development, and Evaluation framework (27). The evaluation was conducted across five domains: risk of bias, inconsistency, indirectness, imprecision, and publication bias. The overall evidence quality for each outcome was rated as high, moderate, low, or very low. This classification system was applied to quantify the strength of the conclusions and to ensure that interpretations were not based on low-quality or unreliable evidence.

## 3 Results

### 3.1 Literature screening

**Figure 1:**
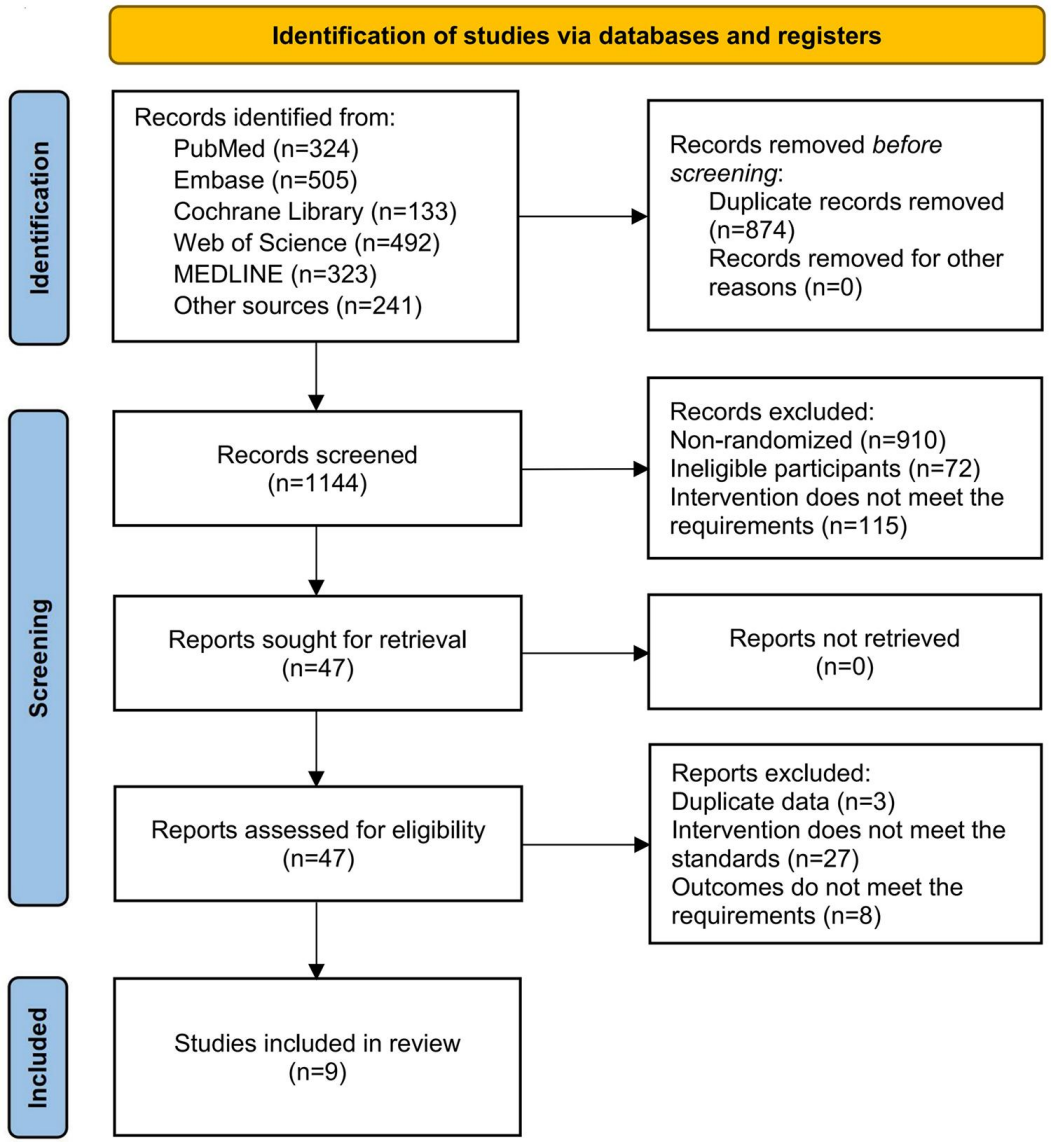
PRISMA flowchartn.

A systematic search across five databases identified a total of 1,777 potentially relevant records. After importing all records into EndNote X9 software (Clarivate Analytics), 633 duplicate articles were removed using the software's automatic detection function combined with manual verification. The remaining records were screened by reviewing titles and abstracts according to the predefined inclusion and exclusion criteria. During this stage, 1,097 articles were excluded, including 910 non-RCRs, 72 studies with ineligible participants, and 115 studies with interventions that did not meet the eligibility criteria. Full-text assessments were subsequently conducted for the remaining 47 articles that met the preliminary inclusion criteria. Of these, 27 articles were excluded due to inconsistencies in intervention protocols, 8 were excluded because of insufficient or irrelevant outcome measures, and 3 were excluded due to data duplication. Ultimately, 9 articles (28–36) were included in the final analysis. The detailed screening and selection process is summarized in Figure 1. The screening checklist is shown in Supplementary Table S1.

### 3.2 Study characteristics

Table 2 summarizes the main characteristics of the nine included studies. Eight studies were conducted in Iran, and one was conducted in China. A total of 478 participants were enrolled, with 245 allocated to the intervention groups and 233 to the control groups. Overall, 38.3% of participants were female, and the mean age across studies was 59.2 years. In all included trials, participants in the intervention groups received probiotic supplementation in addition to standard treatment for coronary heart disease, whereas those in the control groups received standard CHD treatment combined with a placebo. The probiotic regimens varied across studies with respect to strains, dosage, formulation, and administration methods.

**Table 2:** Basic characteristics of included studies.

Study	Country	Sample (E/C)	Female (%)	Age (years)	Route of administration	Intervention	Comparison	Treatment duration (weeks)
Farrokhian et al. (28)	Iran	30/30	63.3	64.1	Take the capsule preparation orally	L. acidophilus, L. casei, and B. bifidum 2 × 10^9^ CFU/day	Placebo	12
Liu et al. (29)	Iran	24/24	35.4	50.5	Take the capsule preparation orally	L.rhamnosus 1.9 × 10^9^ CFU/day	Placebo	8
Moludi et al. (30)	Iran	22/22	6.8	52.6	Take the capsule preparation orally at lunchtime	L. rhamnosus 1.6 × 10^9^ CFU/day	Placebo	12
Moludi et al. (31)	Iran	22/22	6.8	56.9	Take the capsule preparation orally at lunchtime	L.rhamnosus 1.6 × 10^9^ CFU/day	Placebo	12
Moludi et al. (32)	Iran	24/24	35.4	51.5	Take the capsule preparation orally	L. rhamnosus 1.9 × 10^9^ CFU/day	Placebo	8
Raygan et al. (34)	Iran	30/30	/	61.3	Take the capsule preparation orally	B. bifidum, L. casei, and L. acidophilus 2 × 10^9^ CFU/day	Placebo	12
Raygan et al. (33)	Iran	27/27	61.1	63.6	Take the capsule preparation orally	L. acidophilus, L. reuteri, L. fermentum, and B. bifidum 2 × 10^9^ CFU/day	Placebo	12
Sun et al. (35)	China	36/24	43.3	65.6	Take the powder preparation orally	B. lactis 3 × 1010 CFU/day	Placebo	24
Tajabadi et al. (36)	Iran	30/30	/	64.1	Take the capsule preparation orally	L. acidophilus, L. casei, and B. bifidum 2 × 10^9^ CFU/day	Placebo	8

E, experimental group; C, control group; B.bifidum, Bifidobacterium bifidum; B.lactis, Bifidobacterium lactis; L.acidophilus, Lactobacillus acidophilus; L.casei, Lactobacillus casei; L.fermentum, Lactobacillus fermentum; L.rhamnosus, Lactobacillus rhamnosus; L.reuteri, Lactobacillus reuteri. There were no significant differences in female ratio, average age, or disease duration between the experimental and control groups in each included study.

Specifically, in the studies by Farrokhian et al. (28) and Tajabadi et al. (36), participants in the intervention groups received oral probiotic capsules containing Lactobacillus acidophilus, Lactobacillus casei, and Bifidobacterium bifidum at a dose of 2 × 10^9^ CFU/day, while the control groups received matching placebo capsules. Liu et al. (29) and Moludi et al. (32) administered oral probiotic capsules containing Lactobacillus rhamnosus at a dose of 1.9 × 10^9^ CFU/day, with placebo capsules provided to the control groups. In the studies by Moludi et al. (2019) (30) and Moludi et al. (31), participants in the intervention groups received oral probiotic capsules containing Lactobacillus rhamnosus (1.6 × 10^9^ CFU/day), which were explicitly administered at lunchtime; the corresponding control groups received placebo capsules. Raygan et al. (34) administered oral probiotic capsules containing Bifidobacterium bifidum, Lactobacillus casei, and Lactobacillus acidophilus at a dose of 2 × 10^9^ CFU/day, while Raygan et al. (33) used oral probiotic capsules containing Lactobacillus acidophilus, Lactobacillus reuteri, Lactobacillus fermentum, and Bifidobacterium bifidum at the same daily dosage; in both studies, control participants received placebo capsules. In contrast to capsule-based formulations, the study by Sun et al. (35) administered probiotics in powder form, consisting of Bifidobacterium lactis at a dose of 3 × 10^10^ CFU/day, whereas the control group received placebo powder.

Regarding the timing of administration, only the studies by Moludi et al. (30, 31) explicitly reported probiotic intake at lunchtime; the remaining studies did not specify the timing of probiotic administration in relation to meals. With respect to standard CHD treatment, only the study by Sun et al. (35) reported the specific regimen, which included atorvastatin and metoprolol tablets; the other studies did not provide detailed descriptions of standard CHD therapy. The duration of probiotic interventions ranged from 8 to 24 weeks.

### 3.3 Risk of bias

**Figure 2:**
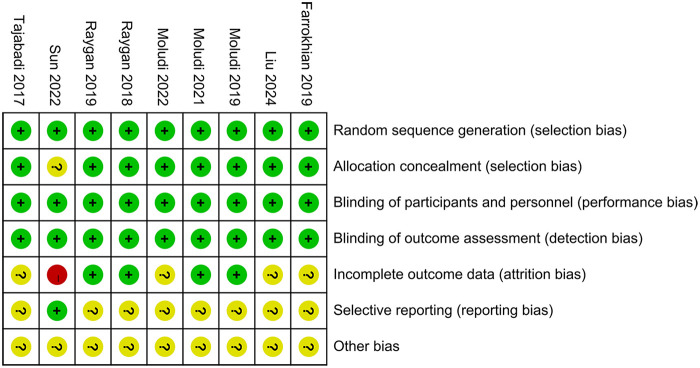
Risk assessment of bias.

The Cochrane Risk of Bias tool 1.0 was applied to assess the methodological quality of the nine included studies (Figure 2). All studies clearly described their methods of random sequence generation and were therefore judged to be at low risk of bias in this domain. One study did not report details regarding allocation concealment and was consequently assessed as having an unclear risk of bias for this item. All included studies appropriately used placebo controls and reported objective outcome measures; accordingly, the risks of performance bias and detection bias were judged to be low across all studies. Regarding attrition bias, four studies reported dropout rates of less than 20% but did not evaluate the potential impact of missing data on the study outcomes; these studies were therefore rated as having an unclear risk of attrition bias. In contrast, one study reported a dropout rate exceeding 20% and was consequently judged to be at high risk of attrition bias. With respect to reporting bias, registration numbers were inaccessible or unavailable for eight studies, leading to an assessment of unclear risk in this domain. Finally, due to insufficient information to identify other potential sources of bias, all studies were judged to have an unclear risk of other bias.

### 3.4 Meta-analysis

#### 3.4.1 Blood lipid-related indicators

**Figure 3:**
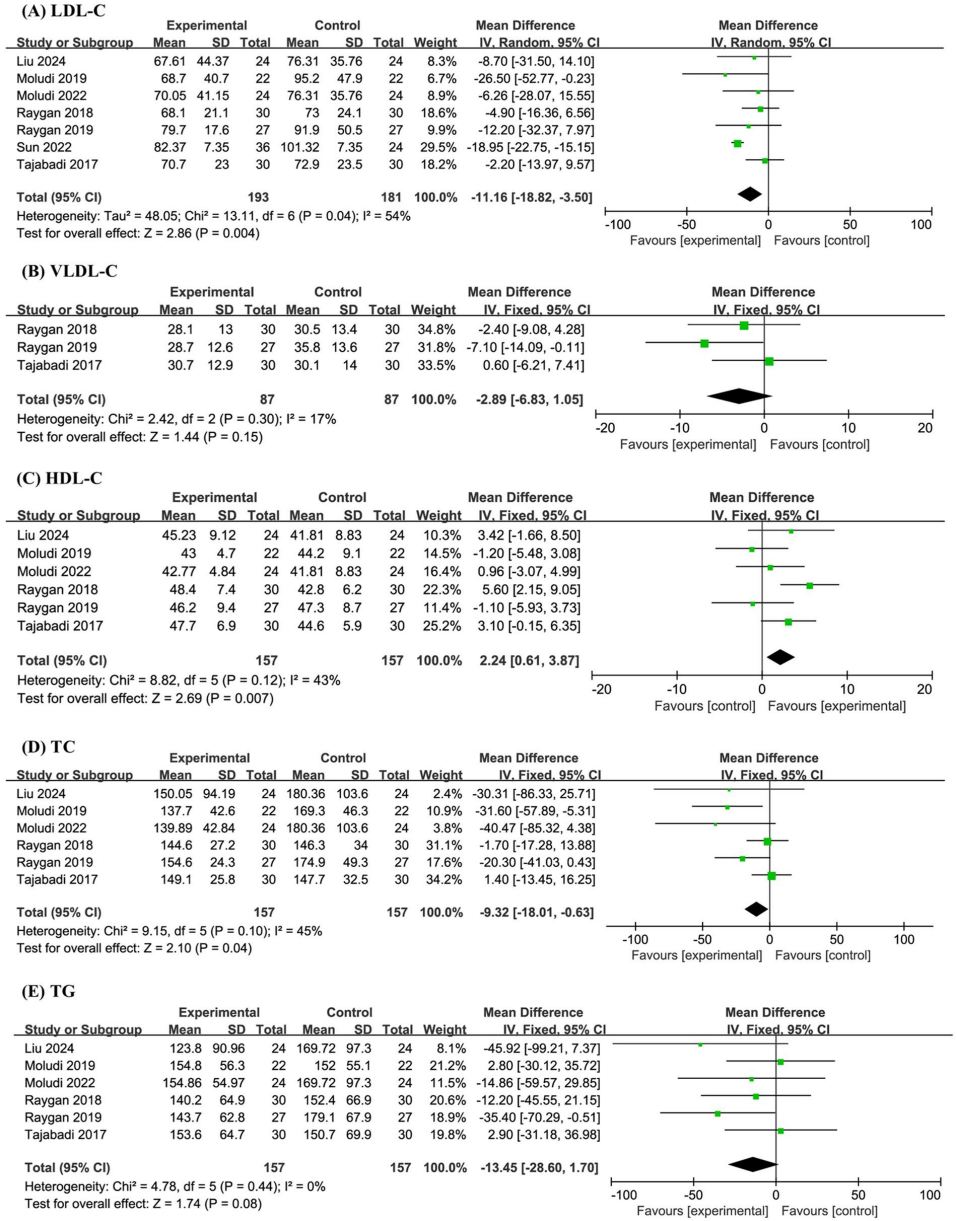
Forest plot of the meta-analysis on blood lipid-related indicators: **(A)** low-density lipoprotein cholesterol (LDL-C); **(B)** very-low-density lipoprotein cholesterol (VLDL-C); **(C)** high-density lipoprotein cholesterol (HDL-C); **(D)** total cholesterol (TC); **(E)** triglyceride (TG).

A total of seven studies involving 374 participants were included in the meta-analysis of LDL-C levels. The results demonstrated that probiotic supplementation significantly reduced LDL-C compared with placebo (MD −11.16, 95% CI −18.82 to −3.50, *P* = 0.004, *I*^2^ = 54%) (Figure 3A).

Three studies with a combined sample size of 174 participants were analyzed for VLDL-C levels. The meta-analysis revealed no significant effect of probiotics on VLDL-C (MD −2.89, 95% CI −6.83 to 1.05, *P* = 0.15, *I*^2^ = 17%) (Figure 3B).

Six studies involving 314 participants were included in the analyses of HDL-C, TC, and TG levels. The pooled results indicated that probiotics significantly increased HDL-C (MD 2.24, 95% CI 0.61 to 3.87, *P* = 0.007, *I*^2^ = 43%), significantly decreased TC (MD −9.32, 95% CI −18.01 to −0.63, *P* = 0.04, *I*^2^ = 45%), and had no significant effect on TG levels (MD −13.45, 95% CI −28.60 to 1.70, *P* = 0.08, *I*^2^ = 0%) (Figures 3C–E).

#### 3.4.2 Blood glucose-related indicators

**Figure 4:**
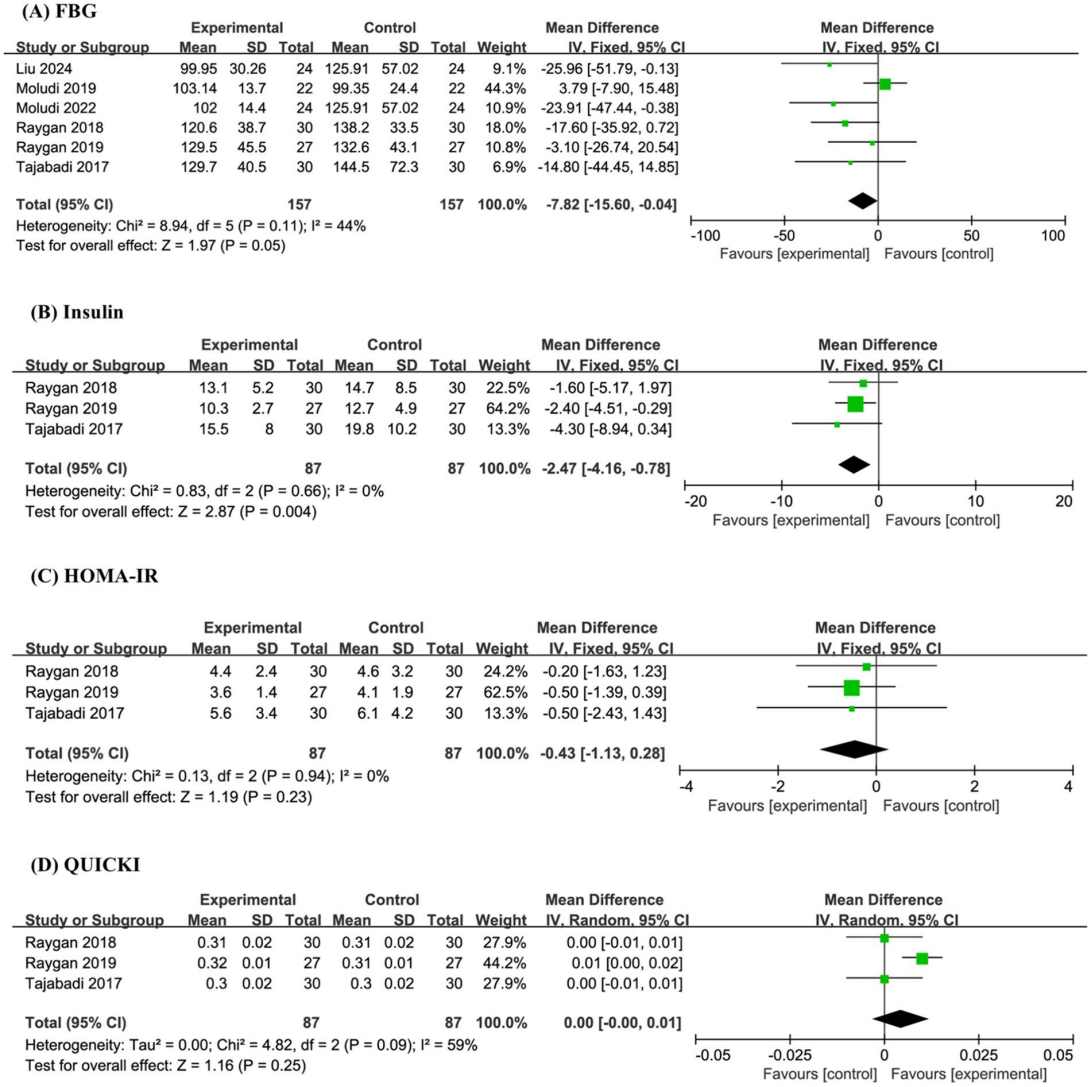
Forest plot of the meta-analysis on blood glucose-related indicators: **(A)** fasting blood glucose (FBG); **(B)** insulin; **(C)** homeostasis model assessment of insulin resistance (HOMA-IR); **(D)** quantitative insulin sensitivity check index (QUICK).

A total of 6 studies and 314 sample sizes conducted targeted analyses on FBG. The meta-analysis revealed that compared with the placebo, probiotics significantly reduced FBG (MD −7.82, 95% CI −15.60 to −0.04, *P* = 0.05, *I*^2^ = 44%) (Figure 4A).

Three studies and 174 sample sizes simultaneously conducted targeted analyses on Insulin, HOMA-IR, and QUICK. The meta-analysis indicated that probiotics significantly reduced Insulin (MD −2.47, 95% CI −4.16 to −0.78, *P* = 0.004, I^2^ = 0%), but had no effect on HOMA-IR (MD −0.43, 95% CI −1.13 to 0.28, *P* = 0.23, I^2^ = 0%) and QUICK (MD 0.00, 95% CI −0.00 to 0.01, *P* = 0.25, *I*^2^ = 59%) (Figures 4B–D).

#### 3.4.3 Blood pressure-related indicators

**Figure 5:**
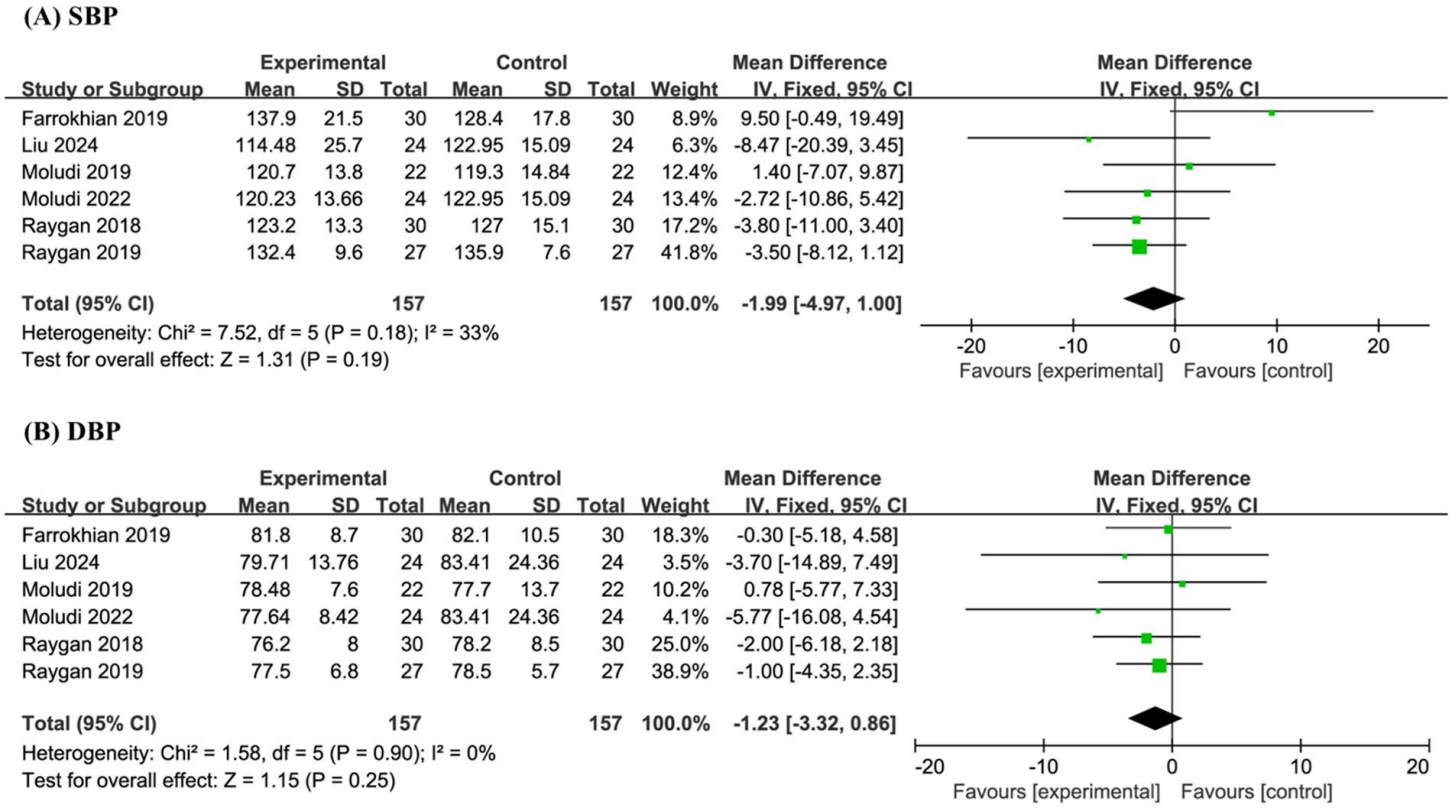
Forest plot of the meta-analysis on blood pressure-related indicators: **(A)** systolic blood pressure (SBP); **(B)** diastolic blood pressure (DBP).

Six studies involving a total of 314 participants were included in the meta-analysis of SBP and DBP. The results indicated that probiotic supplementation had no significant effect on SBP (MD −1.99, 95% CI −4.97 to 1.00, *P* = 0.19, I² = 33%) or DBP (MD −1.23, 95% CI −3.32 to 0.86, *P* = 0.25, *I*^2^ = 0%) when compared to placebo (Figures 5A,B).

#### 3.4.4 Adverse events rate

**Figure 6:**
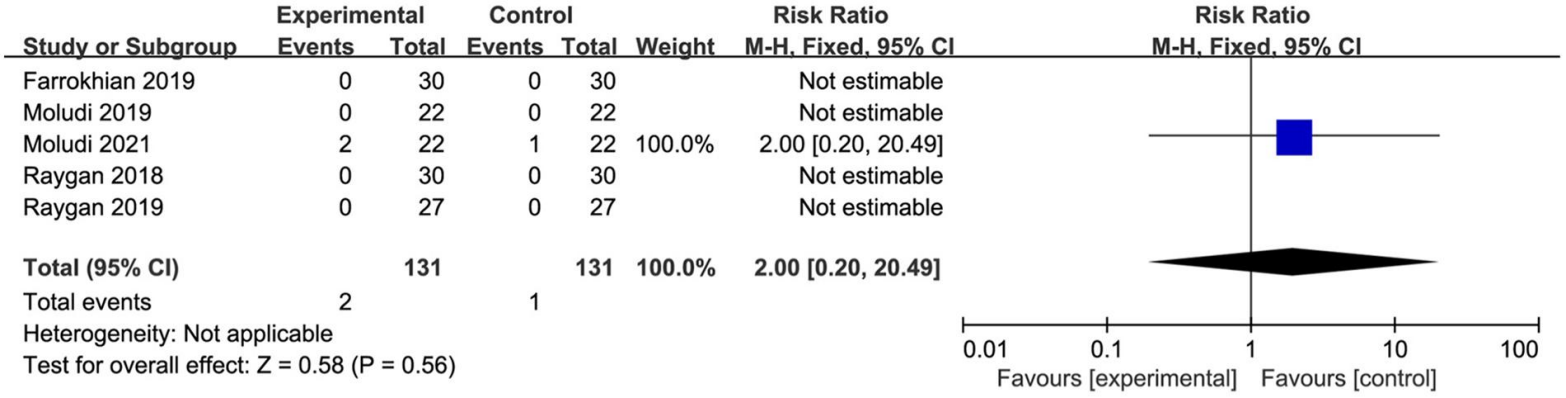
Forest plot of the meta-analysis on adverse events rate.

Five studies involving a total of 262 participants conducted targeted analyses on adverse events. Among these studies, three reported no adverse events, while only Moludi 2021 documented mild gastrointestinal symptoms, including stomach discomfort and gastrointestinal issues, in three participants. The meta-analysis indicated that probiotic supplementation did not significantly affect the incidence of adverse events compared with placebo (MD 2.00, 95% CI 0.20 to 20.49, *P* = 0.56) (Figure 6).

### 3.5 TSA

**Figure 7:**
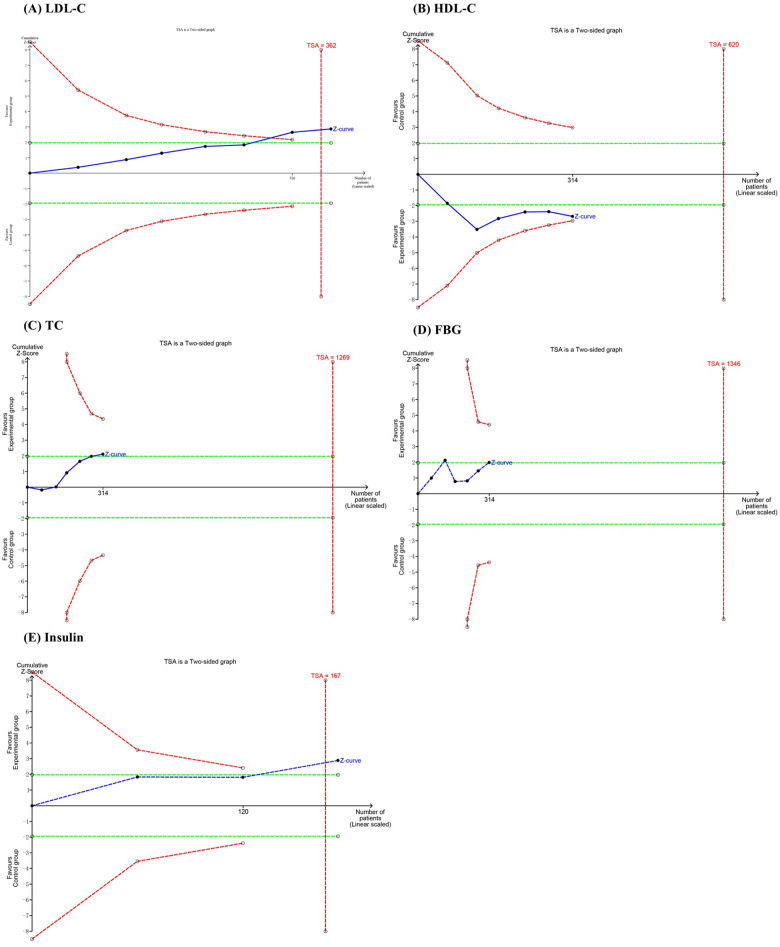
Trial sequential analysis of the efficacy outcomes: **(A)** LDL-C; **(B)** HDL-C; **(C)** TC; **(D)** FBG; **(E)** insulin.

In the TSA, the cumulative Z curves for LDL-C and insulin crossed the monitoring boundary curves, indicating that the meta-analysis results demonstrating the effect of probiotics in reducing LDL-C are statistically robust and conclusive. In contrast, the cumulative Z curves for HDL-C, TC, and FBG did not cross the monitoring boundary curves, suggesting that the current evidence is insufficient and further RCTs are required to confirm the statistical significance of probiotics on these outcomes (Figures 7A–E).

### 3.6 Publication bias

**Figure 8:**
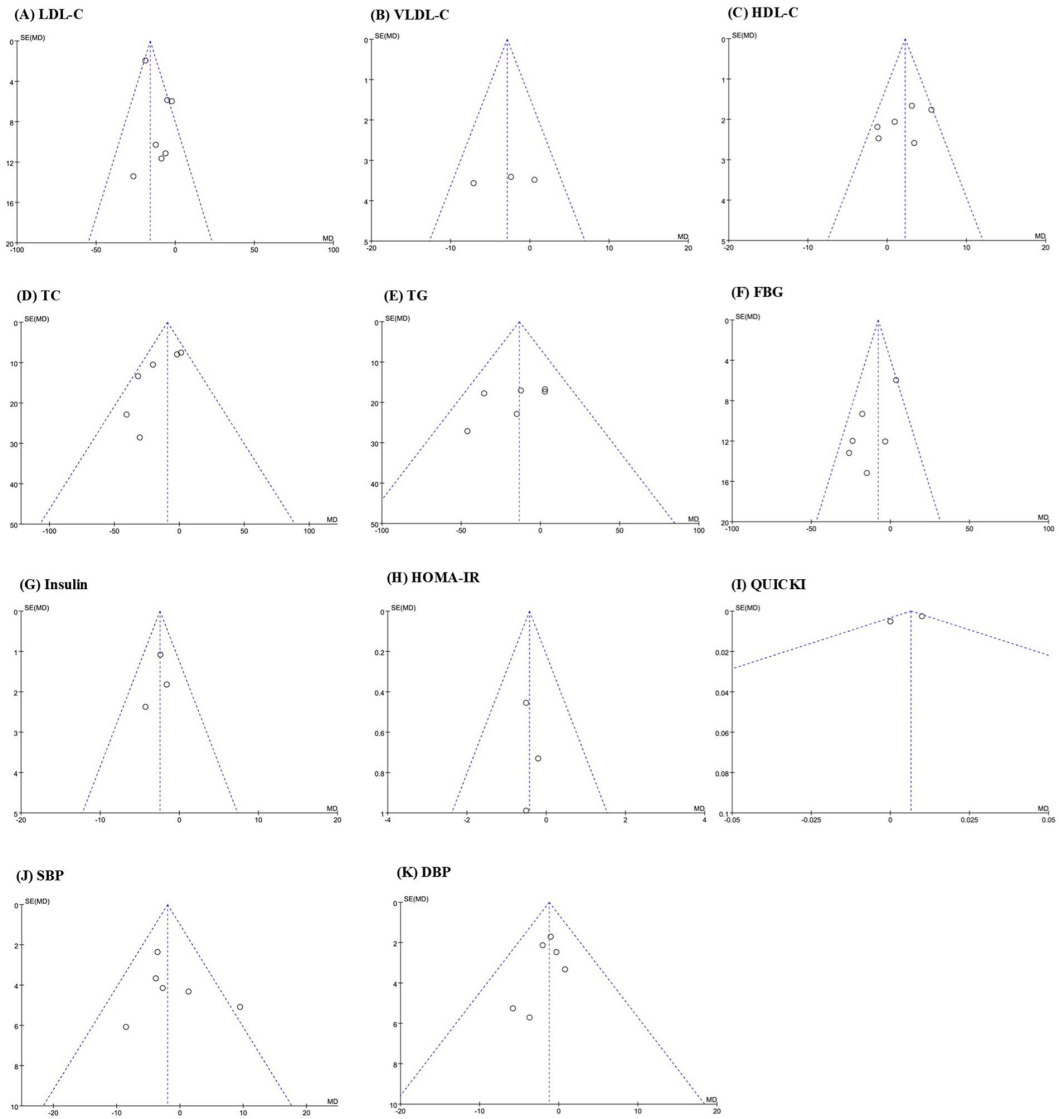
Funnel plot of publication bias: **(A)** LDL-C; **(B)** VLDL-C; **(C)** HDL-C; **(D)** TC; **(E)** TG; **(F)** FBG; **(G)** insulin; **(H)** HOMA-IR; **(I)** QUICK; **(J)** SBP; **(K)** DBP.

Visual analysis of the risk of bias was conducted for 12 outcome indicators. Funnel plots showed that the graphs of VLDL-C, HDL-C, TG, Insulin, HOMA-IR, and QUICK were symmetrically distributed, indicating that there was no publication bias for these indicators. The graphs of LDL-C, TC, FBG, SBP, DBP, and adverse events rate were asymmetric, suggesting that publication bias might exist for these indicators (Figures 8A–K).

### 3.7 Quality of evidence

According to the Grading of Recommendations Assessment, Development, and Evaluation evaluation criteria, VLDL-C, HDL-C, TG, Insulin, and HOMA-IR were classified as moderate-quality evidence, while LDL-C, TC, FBG, QUICK, SBP, DBP, and adverse events rate were classified as low-quality evidence, as shown in Table 3.

**Table 3:** Certainty of evidence.

Outcome	Risk of bias	Inconsistency	Indirectness	Imprecision	Others	RR/MD (95% CI)	Certainty of evidence
LDL-C	None	Serious	None	None	Publication bias	−11.16 (−18.82, −3.50)	Low
VLDL-C	None	None	None	Serious	None	−2.89 (−6.83, 1.05)	Moderate
HDL-C	None	None	None	Serious	None	2.24 (0.61, 3.87)	Moderate
TC	None	None	None	Serious	Publication bias	−9.32 (−18.01, −0.63)	Low
TG	None	None	None	Serious	None	−13.45 (−28.60, 1.70)	Moderate
FBG	None	None	None	Serious	Publication bias	−7.82 (−15.60, −0.04)	Low
Insulin	None	None	None	Serious	None	−2.47 (−4.16, −0.78)	Moderate
HOMA-IR	None	None	None	Serious	None	−0.43 (−1.13, 0.28)	Moderate
QUICKI	None	Serious	None	Serious	None	0.00 (−0.00, 0.01)	Low
SBP	None	None	None	Serious	Publication bias	−1.99 (−4.97, 1.00)	Low
DBP	None	None	None	Serious	Publication bias	−1.23 (−3.32, 0.86)	Low
AER	None	None	None	Serious	Publication bias	2.00 (0.20, 20.49)	Low

RR, risk ratio; MD, mean difference; CI, confidence interval; LDL-C, low-density lipoprotein cholesterol; VLDL-C, very low-density lipoprotein cholesterol; HDL-C, high-density lipoprotein cholesterol; TC, total cholesterol; TG, triglycerides; FBG, fasting blood glucose; HOMA-IR, homeostasis model assessment of insulin resistance; QUICKI, quantitative insulin sensitivity check index; SBP, systolic blood pressure; DBP, diastolic blood pressure; AER, adverse event rate.

## 4 Discussion

### 4.1 Main findings

The role of probiotics and nutrients in metabolism has received increasing attention (37–39). This study performed a meta-analysis and TSA on nine RCTs to systematically evaluate the effects of probiotics on blood lipid and glucose as well as blood pressure in patients with CHD. With respect to blood lipid, probiotics significantly reduced LDL-C and TC levels while increasing HDL-C levels in CHD patients. In terms of blood glucose, probiotic supplementation led to significant reductions in FBG and insulin levels. Regarding blood pressure, however, no significant effects of probiotics were observed on SBP or DBP. TSA confirmed the conclusiveness of the meta-analysis results for LDL-C and insulin. According to the Grading of Recommendations, Assessment, Development and Evaluation assessment, the quality of evidence for these outcomes was rated as Low and Moderate, respectively. These findings indicate that probiotics may hold potential in modulating lipid and glucose metabolism in patients with CHD.

### 4.2 Efficacy and safety analysis

In terms of blood lipid, the probiotic intervention in this study reduced LDL-C by 11.16 mg/dL, increased HDL-C by 2.24 mg/dL, and lowered TC by 9.32 mg/dL in patients with CHD, which aligns closely with both primary and secondary prevention goals for CHD. Notably, TSA confirmed the conclusive nature of the meta-analysis regarding the LDL-C-lowering effect of probiotics. This finding is consistent with *in vitro* studies showing that Lactobacillus reduces cholesterol levels by incorporating cholesterol into its cell membrane and converting it into coprostanol (40), and also corroborates previous meta-analytic evidence indicating that probiotics can regulate blood lipid profiles (41–43). Although the absolute reduction in LDL-C observed in this study did not reach the magnitude typically achieved by statins, probiotics demonstrated a favorable safety profile with no reported adverse effects such as liver damage or myopathy. Therefore, they may serve as a safe alternative or complementary strategy for patients who are unable to tolerate statin therapy. In addition, the increase of HDL-C may exert an anti-atherosclerotic effect by transporting peripheral tissue cholesterol to the liver for metabolism. However, the TSA results suggest that more trials need to be included to determine the robustness of the meta-analysis results, and the same applies to the TC conclusion in the meta-analysis. Despite VLDL-C being a key carrier of TG involved in arterial plaque formation, no significant changes were observed in either TG or VLDL-C levels. This may be attributed to the predominance of Lactobacillus and B. bifidum strains in the included studies, which may not specifically target TG metabolism. Moreover, the relatively short duration of the intervention periods may have limited the potential for gut microbiota remodeling. Given that TG metabolism is more directly influenced by dietary intake of carbohydrates and fats, longer-term interventions may be necessary to observe significant effects.

In terms of blood glucose, probiotics reduced FBG by 7.82 mg/dL and insulin by 2.47 μIU/mL, suggesting that their beneficial effects may be mediated through improved insulin sensitivity. Although no statistically significant changes were observed in HOMA-IR or QUICK, the significant decrease in insulin levels alone indicates a reduced burden on pancreatic β cells, which holds particular clinical relevance for CHD patients with concomitant T2DM. Insulin resistance (IR) is not only a hallmark of T2DM but also a key factor influencing the prognosis of CHD (44). IR contributes to the development of atherosclerotic plaques through multiple mechanisms, including reduced bioavailability of nitric oxide, induction of oxidative stress and inflammatory responses, and promotion of platelet aggregation (45). The capacity of probiotics to ameliorate IR has been extensively supported by experimental and clinical evidence. For instance, Lactobacillus gasseri CKCC1913 improves insulin sensitivity by mediating the enterohepatic axis and facilitating the circulation of SCFAs to the liver, thereby alleviating IR associated with T2DM (46). TSA indicates that the cumulative sample size for insulin has reached the required information size, confirming the robustness of this finding. In contrast, further studies are needed to validate the observed effects on FBG.

There were no significant changes in SBP or DBP, which contrasts with findings from certain studies demonstrating that probiotics can lower SBP and DBP in hypertensive patients (47). Blood pressure regulation is a complex process influenced by multiple physiological systems, including the renin-angiotensin system, sympathetic nervous activity, and vascular endothelial function (48, 49). Probiotics are believed to primarily exert their effects through the gut-vascular axis, and their antihypertensive potential may only manifest in individuals with elevated baseline blood pressure (≥130/85 mmHg) (50). Emerging evidence also suggests that hypertensive patients often exhibit compromised intestinal mucosal barriers and dysbiosis of gut microbiota, characterized by an increased abundance of Gram-negative bacteria and reduced levels of B. bifidum, a genus known for its protective effects on the intestinal barrier (51). In contrast, the study population in this meta-analysis was not restricted by baseline blood pressure status, and therefore, their gut microbiota composition and mucosal barrier integrity may fundamentally differ from those of hypertensive individuals. Although the probiotic formulations used in the included studies contained B. bifidum, their blood pressure-lowering effects may be contingent upon specific pathophysiological conditions, such as the altered gut microenvironment associated with hypertension. Consequently, no significant blood pressure improvements were observed in CHD patients without concomitant hypertension. Lastly, due to the limited sample size, this study was unable to perform subgroup analyses based on baseline blood pressure levels. As a result, potential differential effects across subpopulations could not be assessed, which may partially explain the discrepancy between our findings and those of previous studies.

In terms of safety assessment, only one of the included studies reported three cases of mild gastrointestinal discomfort in the probiotic group, with no instances of study discontinuation due to adverse reactions. According to the aggregated evidence available, probiotic supplementation in patients with CHD demonstrated a favorable safety profile during intervention periods ranging from 8 to 24 weeks, with no significant safety risks directly attributable to the intervention. These findings provide preliminary evidence supporting the short-term safety of probiotic use in CHD patients.

### 4.3 Strain analysis

The included trials involved two single-strain probiotic formulations and three multi-strain formulations. Among the single-strain preparations, L. rhamnosus showed no significant effects on FBG, TG, or HDL-C, and its effects on TC and LDL-C were inconsistent. Specifically, Moludi et al. (30) reported significant reductions in TC (*P* = 0.043) and LDL-C (*P* = 0.049) following L. rhamnosus supplementation; however, these findings were not replicated in a subsequent trial published by the same research group in 2022 (32). This inconsistency suggests that the effects of L. rhamnosus on glucose and lipid metabolism may be negligible or highly unstable. In contrast, another single-strain formulation containing B. lactis was reported to significantly reduce LDL-C levels, although its effects on other glucose- and lipid-related indicators were not investigated (35), which limits the interpretation of its overall metabolic benefits. With respect to multi-strain formulations, the combination of L. acidophilus, L. casei, and B. bifidum significantly reduced insulin and HOMA-β levels but did not significantly affect the primary outcome LDL-C (36), suggesting that its effects may be more pronounced in glucose metabolism than in lipid metabolism. Notably, the remaining two multi-strain formulations demonstrated concurrent regulatory effects on both glucose and lipid metabolism. The formulation composed of B. bifidum, L. casei, and L. acidophilus significantly reduced FBG, insulin, and HOMA-IR levels, while increasing QUICKI and HDL-C (34). Similarly, the formulation containing B. bifidum, L. acidophilus, L. reuteri, and L. fermentum significantly reduced FBG, insulin, HOMA-IR, TG, TC, and VLDL-C levels and increased QUICKI (33). Taken together, these findings suggest that multi-strain formulations containing B. bifidum in combination with multiple Lactobacillus species may represent the most promising probiotic intervention strategies for CHD, as they appear to improve both glucose and lipid metabolism simultaneously.

### 4.4 Mechanism analysis

LDL-C plays a central role in the pathogenesis of CHD. LDL-C deposited in the arterial wall undergoes oxidative modification to form oxidized low-density lipoprotein, which is subsequently phagocytosed by macrophages, thereby initiating and promoting the formation of atherosclerotic plaques (52). Among macrophage subtypes, M1-polarized macrophages internalize lipids to form foam cells and secrete pro-inflammatory cytokines such as interleukin-1β (IL-1β) and tumor necrosis factor-α (TNF-α), which sustain chronic vascular inflammation (53). In contrast, M2-polarized macrophages contribute to fibrous plaque formation following acute endothelial injury (54). Elevated LDL levels are also a common lipid abnormality observed in adolescents with T2DM (55). In contrast to LDL, HDL is often referred to as “good cholesterol” due to its central role in anti-atherosclerosis through reverse cholesterol transport (56). Therefore, lipid-lowering therapy is a critical component in the management of CHD. In addition, glycemic control is equally essential for CHD patients. Hyperglycemia leads to the formation of advanced glycation end products, whose accumulation accelerates the progression of CHD (57). Clinical evidence demonstrates that intensive insulin therapy to control blood glucose in patients with type 1 diabetes can confer cardiovascular benefits lasting up to 30 years (58). Animal studies have further shown that probiotics can reduce atherosclerotic plaque size in murine models (59), with a 12-week supplementation of Lactobacillus rhamnosus being sufficient to achieve this effect (60). The protective effects of probiotics on CHD are not mediated through a single pathway, but rather through a multi-dimensional interaction within the gut-metabolism-immune axis. Based on the findings of this study, we will explore the potential mechanisms underlying the beneficial effects of probiotics on CHD from two perspectives: modulation of gut microbiota composition and suppression of inflammatory responses.

Probiotics exert regulatory effects by reshaping the composition of the intestinal microbiota. On one hand, they delay the progression of CHD by reducing atherosclerosis-promoting metabolites. A key strategy in this regard is the reduction of TMAO levels in the gut. TMAO is generated through the hepatic oxidation of trimethylamine (TMA) by flavin-containing monooxygenases, and the TMA-TMAO metabolic pathway serves as a critical link between gut microbiota and CHD pathogenesis. Upon ingestion of choline-, L-carnitine-, or betaine-rich foods, these compounds are metabolized by specific microbial enzymes in the intestine, such as choline-TMA lyase and carnitine monooxygenase, to produce TMA. TMA is then absorbed into the portal circulation, converted to TMAO in the liver, and ultimately excreted via urine (61). TMAO exerts multiple detrimental effects on the cardiovascular system. First, it activates reactive oxygen species and induces lysosomal rupture, which triggers the activation of Nucleotide-binding oligomerization domain, leucine-rich repeat and pyrin domain-containing 3 inflammasomes in endothelial cells and promotes the release of pro-inflammatory cytokines IL-1β and interleukin-18, ultimately leading to endothelial dysfunction (62). Second, TMAO enhances the expression of the oxidized low-density lipoprotein receptor cluster of differentiation 36 via the Mitogen-Activated Protein Kinase/c-Jun N-Terminal Kinase signaling pathway, resulting in cholesterol accumulation and foam cell formation, thereby accelerating AS progression (63). Finally, TMAO also promotes myocardial fibrosis by upregulating fibrotic mediators such as collagen through the Janus Kinase 2/Signal Transducer and Activator of Transcription 3 pathway (64). On the other hand, probiotics can inhibit CHD progression by increasing the production of protective metabolites such as SCFAs. SCFAs exert anti-atherosclerotic effects primarily through the suppression of inflammatory responses (16, 65). SCFAs include acetic acid, propionic acid, butyric acid, isobutyric acid, valeric acid, isovaleric acid, and caproic acid, with propionic acid and butyric acid being the most extensively studied. In animal experiments, Arash Haghikia et al. demonstrated that propionic acid reduces LDL-C levels by upregulating Interleukin-10 expression and inhibiting the activation of the cholesterol transporter Niemann-Pick C1-like 1 (66). This finding was further supported by clinical evidence showing that patients with high cholesterol exhibited significant reductions in LDL-C and TC levels after an 8-week oral supplementation of propionic acid. These results suggest that elevated propionic acid levels confer protective benefits for CHD patients. Additionally, *in vitro* studies have shown that butyric acid can delay the progression of atherosclerotic lesions by suppressing cluster of differentiation 36 expression in macrophages and reducing IL-1β production (67).

Chronic inflammation serves as both a central pathogenic driver in the development and progression of CHD and a core mechanism underlying IR in diabetes (44). Probiotics exert their anti-inflammatory effects primarily through two mechanisms: restoration of the intestinal barrier and modulation of intestinal immune cell function. Intestinal barrier dysfunction is commonly observed in CHD patients. Increased intestinal mucosal permeability allows LPS to translocate from the gut lumen into the systemic circulation (68). Once in the bloodstream, LPS activates Toll-like receptor 4, initiating downstream signaling via Nuclear Factor-κB (NF-κB) and Mitogen-Activated Protein Kinase pathways, which leads to the production of pro-inflammatory mediators (69). Concurrently, LPS-induced signaling inhibits the phosphorylation of insulin receptor substrate-1, thereby impairing insulin signaling and promoting IR (70). Enhanced activation of the Nucleotide-binding oligomerization domain, leucine-rich repeat and pyrin domain-containing 3 inflammasome has been observed in patients with T2DM, accompanied by elevated levels of its downstream pro-inflammatory cytokines IL-1β and interleukin-18 (71). During the progression of chronic inflammation, neutrophil extracellular trap formation (NETosis) has emerged as a key pathological process that directly links CHD with diabetes (72). Neutrophil extracellular traps are web-like chromatin structures released by activated neutrophils, and NETosis represents a crucial component of the innate immune response (73). Neutrophil extracellular traps promote endothelial dysfunction by activating endothelial cells, thereby accelerating the development of AS, as confirmed in animal models (74, 75). Notably, circulating levels of NETosis markers are significantly elevated in patients with T2DM (76), further supporting its role as a potential therapeutic target in individuals with comorbid diabetes and CHD (72). Zhujun An et al. demonstrated in both *in vitro* and *in vivo* models that interleukin-18-induced NETosis exacerbates AS through activation of the NF-κB signaling pathway (77). In animal studies, supplementation with Lactobacillus acidophilus ATCC4356 in apolipoprotein E knockout mice was shown to attenuate inflammation by inhibiting NF-κB activation, resulting in reduced TNF-α levels and increased Interleukin-10 production, thereby improving the overall inflammatory profile (78). TNF-α exacerbates inflammation by compromising vascular barrier integrity, whereas Interleukin-10 exerts protective effects by suppressing the release of pro-inflammatory cytokines (79). Similarly, daily administration of Lactobacillus plantarum ATCC14917 was found to decrease serum levels of IL-1β and TNF-α while reducing the degradation of inhibitor of nuclear factor kappa-B alpha, a key inhibitory protein in the NF-*κ*B pathway, thereby exerting anti-inflammatory effects (80). Further experimental evidence from studies using apolipoprotein E knockout mice supplemented with Lactobacillus rhamnosus for 8 weeks revealed significant reductions in TNF-α, monocyte chemoattractant protein-1, and Interleukin-6 levels (51). Interleukin-6 stimulates hepatic production of C-reactive protein, contributing to systemic vascular inflammation, while monocyte chemoattractant protein-1 facilitates monocyte recruitment to sites of vascular injury, promoting plaque progression (81). The observed reductions in these inflammatory mediators are strongly associated with decreased CHD risk. Collectively, these findings indicate that specific probiotic strains can mitigate vascular endothelial damage and slow the progression of atherosclerosis by modulating inflammatory cytokine profiles and restoring balance to the vascular inflammatory microenvironment. This represents a key mechanism underlying the anti-inflammatory protective effects of probiotics in CHD.

### 4.5 Limitations and perspectives

Several limitations of this study should be acknowledged. First, the number of eligible studies included in the quantitative synthesis was relatively small (*n* = 9). Although additional searches and repeated screening were conducted during the revision process, no further studies meeting the predefined inclusion criteria were identified. This reflects the current scarcity of RCTs specifically investigating probiotic supplementation in patients with CHD, rather than selective inclusion by the authors. Consequently, while the available evidence was synthesized rigorously, the limited number of studies may reduce the statistical power for detecting modest effects and precluded more detailed subgroup analyses.

Second, the generalizability of the findings is limited by the geographical concentration of the included studies. Eight of the nine trials were conducted in Iran, with only one study from China, and no eligible data from Europe, North America, or other regions. Beyond simple geographic representation, interregional differences in habitual diet, host genetics, and healthcare systems may modulate both the baseline state of the gut microbiota and host responses to probiotic supplementation. Dietary patterns vary substantially across populations, and such variation has been shown to influence gut microbial composition and metabolic outputs. For example, plant-rich or Mediterranean-style diets that are high in fiber and polyphenols have distinct effects on gut microbial taxa and associated metabolites compared with Western high-fat, high-saturated fat diets, and these differences have been linked to variation in cardiometabolic risk profiles (82, 83). Moreover, host genetic background may also shape gut microbiota composition and modulate metabolic responses. Emerging evidence from multi-omics studies indicates complex interactions among diet, host genome, and gut microbiome that contribute to variability in cardiovascular disease risk and metabolic phenotypes (84). Finally, differences in healthcare systems and standard CHD management protocols, including the availability and use of lipid-lowering agents, antihypertensives, and guideline-directed medical therapy, could alter the background against which probiotic supplements exert any additional effects (85, 86). Collectively, these factors underscore the need for future randomized controlled trials conducted in diverse settings to determine whether the effects observed in the current studies can be replicated across different dietary, genetic, and healthcare contexts.

Third, the present analysis primarily focused on intermediate metabolic markers, including blood lipids and glycemic parameters, rather than hard clinical endpoints. Although these surrogate outcomes are biologically relevant and commonly used in early-stage intervention studies, they do not necessarily translate into reductions in cardiovascular events, mortality, or atherosclerotic plaque progression. The lack of data on clinically meaningful outcomes represents a major gap in the current evidence base. Future RCTs should prioritize the inclusion of cardiovascular events, disease progression, and long-term prognosis as primary outcomes, in order to establish whether probiotic supplementation provides tangible clinical benefits for patients with CHD.

Overall, addressing these limitations through well-designed, large-scale, and geographically diverse clinical trials with clinically relevant endpoints will be essential to clarify the role of probiotics as a complementary strategy in the prevention and management of coronary heart disease.

## 5 Conclusion

Probiotic supplementation demonstrates significant efficacy in reducing LDL-C and insulin levels among patients with CHD without increasing the risk of adverse events. These findings provide strengthened evidence supporting the potential role of probiotics as an adjunctive strategy in CHD management. However, the current evidence is primarily derived from intermediate metabolic markers rather than hard clinical outcomes. Future well-designed, large-scale randomized controlled trials incorporating hard clinical outcomes are therefore essential to determine whether improvements in surrogate metabolic markers translate into tangible clinical benefits for patients with CHD.

## Data Availability

The original contributions presented in the study are included in the article/Supplementary Material, further inquiries can be directed to the corresponding author.
