# Supplementary material for: Effects of probiotics on blood lipids, glucose and pressure in patients with coronary heart disease: a systematic review and meta-analysis
Source: Front Cardiovasc Med. 2026 Feb 6;13:1707408. doi: 10.3389/fcvm.2026.1707408 (PMC12920485; doi:10.3389/fcvm.2026.1707408)
Supplement: Supplementary file 1 [file Table1.docx]

**Supplementary Table 1** PICOS-based study screening checklist.

| Domain | Screening question | Eligibility criteria | Decision (Yes/No/Unclear) |
| --- | --- | --- | --- |
| Participants (P) | Are the participants adult patients diagnosed with CHD? | Adults (≥18 years) with clinically diagnosed CHD |  |
| Intervention (I) | Does the study involve probiotic supplementation combined with standard CHD treatment? | Any probiotic strain, dose, or formulation added to standard therapy |  |
| Comparison (C) | Is the control group treated with standard CHD therapy alone or placebo? | Placebo or standard treatment without probiotics |  |
| Outcomes (O) | Are predefined outcomes reported? | LDL-C*, HDL-C, TC, TG, VLDL-C, FBG, insulin, HOMA-IR, QUICK, SBP, DBP, adverse events |  |
| Study design (S) | Is the study a randomized controlled trial? | Parallel or crossover randomized controlled trials |  |
| Data completeness | Are outcome data sufficient for quantitative synthesis? | Means, SDs, change values, or extractable data available |  |
| Duplicate/Overlap | Is the study free from duplicate or overlapping data? | No duplicated population or repeated publication |  |

CHD, coronary heart disease. * means the primary outcome.
